# Marriage in England

**Published:** 1877-10

**Authors:** 


### MARRIAGE IN ENGLAND.

THE indifference and even contempt with which the marriage tie is too often treated in the United States, is producing its natural fruit. The manner in which it is protected in England furnishes an example, in some respects, which we might advantageously follow —especially as it relates to the rites of what is absurdly called the solemnization of marriage by our petty magistrates, without the possibility of their knowing whether the parties are not already guilty of bigamy. Our popular journals, which are fond of gathering up all the details of crim. con., divorce, and elopement, bear a sad testimony to the little respect paid to conjugal obligations. "Wife-murder" is now an established heading in the tragic column of our city papers.

The different modes of marriage allowed in England and Wales are as follows:

1. According to the rites of the Established Church.

2. In registered places of worship, belonging to the Dissenting bodies, Roman Catholics, etc.

3. In the District Register's office.

4. Among the Quakers and Jews, according to their respective "usages."

Under each of these heads, there are certain forms required. In the Church of England, there may be granted: 1. A special license, which can be granted only by the Archbishop of Canterbury, and which permits the marriage "at any convenient time and place." This costs a heavy fee, and there is not more than an average of eleven marriages under it in a year. 2. The common ecclesiastical license granted by a surrogate for marriage in the parish church, or in some public chapel, within the jurisdiction of which one of the parties must have resided for at least fifteen days. The marriage may follow immediately on the granting of the license. Without any previous notice, the parties may apply and be married within the hour. 3. For marriage after the publication of bans, they must be proclaimed on three Sundays in the parish church, and if they reside in different parishes, in the church of each.

Among Dissenters, marriage may be solemnized either by license or by certificate, both of which must be obtained from the Superintendent Registrar. Notice, in prescribed form, must be given to that officer, who records it in a "notice book," which is open for inspection one whole day, after which the license is granted. Either of the parties may obtain it, but one of them must have resided fifteen days in the district. Notice in one district is sufficient, where they have different residences.

The marriage may be solemnized at the usual place of worship of one of the parties, outside of the district, but within two miles of it, and they may go into another district, when there is no registered place of worship in their own. The same freedom is allowed with respect to marriages in Dissenting chapels by certificate, in which case the notice thereof must be affixed in the Registrar's office for twenty-one days after the record of the particulars in the "notice" marriage book; and then the certificate may issue. Only seven days' residence is necessary before giving notice of marriage without license.

Marriages are performed in the Register's office on the production of the license or certificate. The presence of the Superintendent Registrar, as well as that of a Registrar of marriages, is requisite, and both officers must sign the register.

No marriage by certificate can be solemnized in a church or chapel without the consent of the officiating minister.

Jews may marry with or without license, either in a synagogue or private dwelling, and at any hour of the day; the Quakers also without license, in one of their meeting-houses, between the hours of eight and twelve.

The reason why Quakers (and the same, probably, with Jews) are exempted from the legal forms required for other sects, is that their own forms, which constitute a part of their religious system, and to the observance of which they are bound by conscientious scruples, as well as by their internal discipline, fully supply all the guards and requirements of the law.

